# 

**Published:** 1890-05-24

**Authors:** 


SATURDAY, MAY 24th, 1890.

**Charitable Trusts and Charity Commissioners** . . . 101

**Words of Consolation.**—Weary Nights . . . 102

**The Doctor's Pulpit.**—Cremation or "Decent Burial"? . . 103

**Working Women in Large Towns.** III.—Factory Girls 104

**Before the Lords' Committee** (By our Special Commissioner) 105

**Everybody's Page** . . . . . . 107

**Notes and News** . . . . . . 108

**Annotations.**—A Sanitary Duel at Sheffield.—Is She Not a Nurse?—A Summer Duty . . . 110

**The Practitioner's Mirror and Retrospect:**

MEDICINE—Myxœdema . . . . . 111

SURGERY—Progress of Surgery . . . . 111

THERAPEUTICS—Cannabis Indica . . . . 112

THE PRACTITIONER'S BOOKSHELF—Leçons de Gynécologie Opératoire—The Diseases and Disorders of the Ox . . 113

**Passing Topics.**—The London Post-Graduate Course . . 114

**Scraps and Gleanings!** . . . . . 114

**The Month of May in India** . . . . 114

EXTRA SUPPLEMENT—THE NURSING MIRROR.

**En Passant.**—Queen Charlotte's Hospital—One of the Elite—Short Items—Rural Nursing Association—The Metropolitan Association—Arrangements for June . . xxix
**Nurse Joyce.** Part I. . . . . . xxx
**The Nurses' Bookshelf.**—Antiseptics for Nurses—Food in Motherhood . . . . . xxxi
**Presentations** . . . . . . xxxi
**Everybody's Opinion.**—A Holiday Home—Metropolitan and National District Nurses—Junius S. Morgan Memorial xxxi
**Appointments** . . . . . . xxxii
**The National Pension Fund for Nurses** . . . xxxii
**Notes and Queries** . . . . . xxxii
**Vacancies** . . . . . . xxxii
**Amusements and Relaxation** . . . . xxxii

